# Systematic review of the association between talc and female reproductive tract cancers

**DOI:** 10.3389/ftox.2023.1157761

**Published:** 2023-08-07

**Authors:** Heather N. Lynch, Daniel J. Lauer, Olivia Messina Leleck, Rachel D. Freid, Justin Collins, Kathleen Chen, William J. Thompson, A. Michael Ierardi, Ania Urban, Paolo Boffetta, Kenneth A. Mundt

**Affiliations:** ^1^ Stantec (ChemRisk), Boston, MA, United States; ^2^ Stantec (ChemRisk), Boulder, CO, United States; ^3^ Stantec (ChemRisk), Pittsburgh, PA, United States; ^4^ Stantec (ChemRisk), Brooklyn, NY, United States; ^5^ Stantec (ChemRisk), San Francisco, CA, United States; ^6^ Stony Brook Cancer Center, Stony Brook, NY, United States; ^7^ Department of Medical and Surgical Sciences, University of Bologna, Bologna, Italy

**Keywords:** systematic review, talc, hazard assessment, ovarian cancer, uterine cancer

## Abstract

Talc is a hydrous magnesium sheet silicate used in cosmetic powders, ceramics, paints, rubber, and many other products. We conducted a systematic review of the potential carcinogenicity of genitally applied talc in humans. Our systematic review methods adhere to Preferred Reporting Items for Systematic Reviews and Meta-Analyses (PRISMA) guidelines and incorporated aspects from the US Institute of Medicine (IOM, now the National Academy of Medicine) and several US EPA frameworks for systematic reviews, evaluating and integrating the epidemiological, animal, and mechanistic literature on talc and cancer. We conducted a comprehensive literature search. Detailed data abstraction and study quality evaluation, adapting the Toxic Substances Control Act (TSCA) framework, were central to our analysis. The literature search and selection process identified 40 primary studies that assessed exposure to talc and female reproductive cancer risks in humans (n = 36) and animals (n = 4). The results of our evaluation emphasize the importance of considering biological plausibility and study quality in systematic review. Integrating all streams of evidence according to the IOM framework yielded classifications of *suggestive evidence of no association* between perineal application of talcum powders and risk of ovarian cancer at human-relevant exposure levels. We also concluded that there is *suggestive evidence of no association* between genital talc application and endometrial cancer, and *insufficient evidence to determine whether a causal association exists* between genital talc application and cervical cancer based on a smaller but largely null body of literature.

## Introduction

Talc is a naturally occurring, inert hydrous magnesium silicate mineral with the chemical formula Mg_3_Si_4_O_10_(OH)_2_. Extracted talcs contain pure talc as well as a variety of potential accessory minerals. Pharmaceutical-grade talc is the purest (minimum of 99% talc) followed by cosmetic-grade talc and industrial-grade talc (Fiume et al., 2015). Cosmetic and pharmaceutical talcs are required to have no detectable fibrous minerals, but industrial talc may contain a variety of accessory minerals including tremolite, anthophyllite, carbonate and a small amount of crystalline silica. It has been reported that some cosmetic talcs as well as finished talcum powders may have contained trace levels of asbestiform minerals despite the lack of evidence of asbestos at some major talc sources (Ciocan et al., 2022). This disconnect may stem from challenges with accurately identifying and quantifying asbestiform minerals in talc (Fiume et al., 2015; Pierce et al., 2017). As a result, there remains uncertainty regarding the composition of the talcs to which populations in epidemiological studies have been exposed.

Talc-containing powders, or talcum powders, have been used for feminine hygiene purposes for decades (IARC, 2010). In the 1980s and 1990s, concerns over the potential carcinogenicity of talc emerged following the first epidemiological study of ovarian cancer risk and a National Toxicology Program (NTP, 2019) rodent carcinogenicity study (Fiume et al., 2015). Subsequently, a large body of epidemiological literature has investigated the purported association between perineal talc use and female reproductive cancers, largely ovarian cancer. In 2010, International Agency for Research on Cancer (IARC) evaluated epidemiological findings from one prospective cohort study and 19 case-control studies and concluded, “[t]here is limited evidence in humans for the carcinogenicity of perineal use of talc-based body powder” (IARC, 2010). Similarly, most but not all recent reviews on talc exposure and reproductive cancer risk have found limited but inconsistent evidence of an association for ovarian cancer (Kadry Taher et al., 2019; Goodman et al., 2020; Wentzensen and O’Brien, 2021; Micha et al., 2022). However, these reviews are generally narrative but not formal systematic reviews, and most examined only epidemiological evidence.

Regarding the potential translocation of talc within the female reproductive system, it is important to note that talc varies in its physicochemical properties, including particle size and shape, which affects its disposition in the body and overall toxicity. Cosmetic talc particle sizes vary by product type and have reportedly ranged from 4 to 15 µm (Fiume et al., 2015). Given that talc is an insoluble solid, it is poorly absorbed in the gastrointestinal mucosa, and dermal absorption through intact skin is not expected to occur (Health Canada Screening Assessment Talc, 2021). Some studies have indicated that smaller talc particle sizes may be able to translocate readily across the pleura and into the bloodstream after intrapleural injection, but there is little evidence that talc readily translocates to other areas of the body after inhalation into the lungs (Rossi et al., 2010; Fiume et al., 2015). As such, for talc to be associated with ovarian and other reproductive cancers, talc would first need to migrate from the respiratory tract into general circulation then to the reproductive organs (a pathway with little to no evidence (ECHA, 2021; Wehner, Zwicker, and Cannon, 1977)), or from the perineal area outside the body, into the vagina, through the uterus, fallopian tubes, and into the ovary (i.e., translocation, possibly via smooth muscle contraction and other mechanisms that move ova and spermatozoa). Second, there would need to be plausible Mode of action (MOA) whereby the talc possibly reaching the reproductive tract induces carcinogenic processes, such as inflammatory responses in these tissues.

Given the inconsistent associations observed in the epidemiological literature and the lack of mechanistic data implicating talc carcinogenicity, we sought to perform a comprehensive systematic review of the current body of literature, utilizing all lines of evidence and following standard systematic review principles. The primary objective of this systematic review was to critically evaluate the possible relationship(s) between perineal exposure to talc-containing products (primarily talcum powders and cosmetics) and female reproductive tract cancer(s), critically assessing and integrating evidence from epidemiology, toxicology, and studies informing potential underlying MOAs.

## Materials and methods

This systematic review was conducted in accordance with the Preferred Reporting Items for Systematic Reviews and Meta-Analyses (PRISMA) checklist, using a hybrid systematic review framework, incorporating aspects from several recognized systems. Specifically, we relied most heavily on the United States Environmental Protection Agency’s (EPA) protocol for systematic reviews conducted under the Toxic Substances Control Act (TSCA) and the Draft Handbook for the Integrated Risk Information System (EPA, 2005). Hazard conclusions were guided by the US Institute of Medicine (IOM, now National Academy of Medicine) framework (IOM, 2009). An overview of our methods is provided below, and a more detailed description can be found in our prior evaluation of pulmonary cancers (Lynch et al., 2022) and in the supplemental Protocol, allowing verification and replication of our review.

**FIGURE 1:**
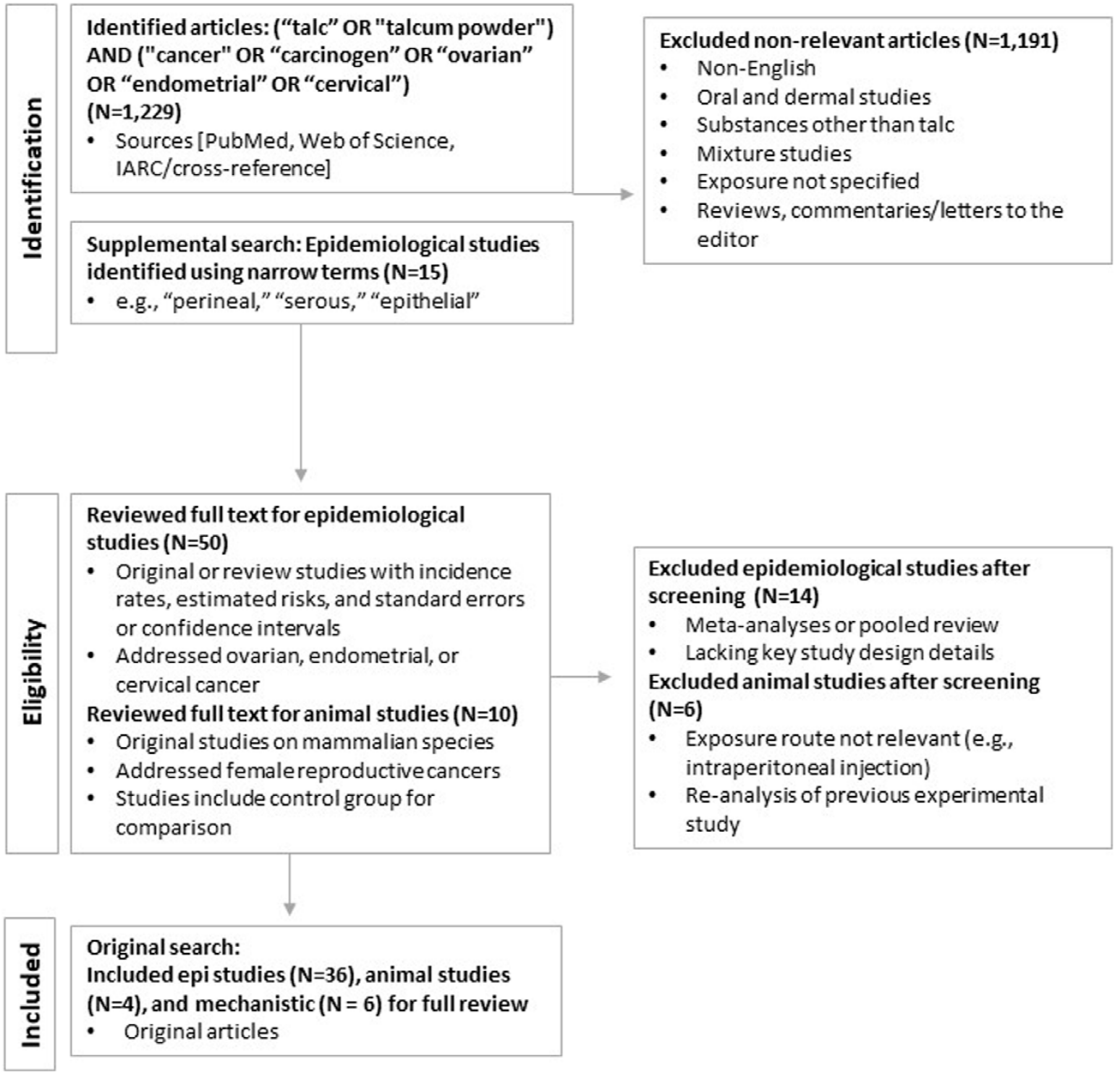
Literature search and selection process.

In brief, we performed literature searches using PubMed and Web of Science using broad search terms for any cancer type, cross-referencing with existing agency reviews, including IARC (see Protocol provided in the Supplementary Materials for additional detail). Studies were selected using *a priori* inclusion and exclusion criteria specific to each realm of evidence, including epidemiological studies, experimental animal studies in mammalian species, and mechanistic studies *in vivo* or in mammalian or bacterial cell lines (see Figure 1). This review focuses on talc exposure via the perineal and related (e.g., via diaphragms and intrauterine devices [IUDs]) routes.

Each study meeting inclusion criteria underwent full data extraction and was further evaluated for methodological quality using a modified version of the study quality framework used in US EPA TSCA risk evaluations (EPA, 2018; updated in EPA, 2021). For epidemiological studies, we employed qualitative, tiered approaches specific to study design. The tiering system allowed for preferentially weighting specific quality domains (e.g., exposure characterization) addressing key sources of potential bias first, and then secondary determinants of study quality. Flow charts of the overall tiering approaches are provided in Supplementary Figures S1 and S2.

Evidence was synthesized for each individual line of evidence and then integrated to reach conclusions regarding potential carcinogenicity, considering consistency, coherence and exposure-response relationships, if any. Overall conclusions were derived for each cancer based on the following IOM classifications for causation: sufficient evidence of a causal relationship; sufficient evidence, limited/suggestive evidence, or inadequate/insufficient evidence of an association; or limited/suggestive evidence of no association (IOM, 2009) (Table 1).

**TABLE 1:** IOM (2001) Categorizations for evaluating strength of evidence^a^

Classification	Description
Sufficient Evidence of a Causal Relationship	Evidence is sufficient to conclude that a causal relationship exists between the exposure to a specific agent and a health outcome in humans. The evidence fulfills the criteria for sufficient evidence of an association (below) and satisfies several of the criteria used to assess causality: strength of association, dose-response relationship, consistency of association, temporal relationship, specificity of association, and biological plausibility
Sufficient Evidence of an Association	Evidence is sufficient to conclude that there is a positive association. That is, a positive association has been observed between an exposure to a specific agent and a health outcome in human studies in which chance, bias, and confounding could be ruled out with reasonable confidence
Limited/Suggestive Evidence of an Association	Evidence is suggestive of an association between exposure to a specific agent and a health outcome in humans, but is limited because chance, bias, and confounding could not be ruled out with confidence
Inadequate/Insufficient Evidence to Determine Whether an Association Does or Does Not Exist	The available studies are of insufficient quality, consistency, or statistical power to permit a conclusion regarding the presence or absence of an association between an exposure to a specific agent and a health outcome in humans
Limited/Suggestive Evidence of No Association	There are several adequate studies covering the full range of levels of exposure that humans are known to encounter, that are mutually consistent in not showing a positive association between exposure to a specific agent and a health outcome at any level of exposure. A conclusion of no association is inevitably limited to the conditions, levels of exposure, and length of observation covered by the available studies. In addition, the possibility of a very small elevation in risk at the levels of exposure studied can never be excluded

Source: IOM, 2009.

^a^ IOM has since updated the classification language, but the same general underlying considerations are used for reaching each conclusion. The previous classification categories were retained as we believed the previous categories were more easily interpreted than the updated categories.

## Results

### Literature search and selection

The primary literature search in PubMed (updated as of December 2022) yielded a total of 1,229 publications. After eliminating duplicate entries or studies that were subsequently updated and applying the inclusion and exclusion criteria, 36 epidemiological and four animal studies remained and were selected for review. Additional searches in Web of Science identified no additional publications. The results of the literature search and study identification and selection are summarized in Figure 1. Ultimately, the relevant literature addressed three cancers of the female reproductive tract: ovarian, cervical, and endometrial.

### Animal and human studies evaluating talc translocation to the reproductive system

Four experimental animal studies evaluated the potential for talc externally applied or introduced vaginally to reach the internal reproductive organs: these studies are summarized in brief below. Unfortunately, the four identified studies did not provide comprehensive details regarding the physiochemical nature of the talc used. Thus, it was not possible to elucidate the effect of variation in physiochemical talc properties on the biological effects observed.

Phillips et al. (1978) administered a single intravaginal dose of 0.5 mL of 3H-labelled talc to three rabbits and six consecutive daily intravaginal doses of 0.5 mL 3H-labelled talc to another group of three rabbits. The animals were sacrificed 3 days later and urine, ovary, fallopian tube, endometrium and cervix, vagina and bladder samples were collected. No translocation of talc to the ovaries was observed.

Wehner et al. (1985) administered 125 mg of a neutron-activated cosmetic talc blend (Johnson’s baby powder) suspended in 0.3 mL of deionized water containing 1% carboxymethyl cellulose vaginally to two cynomolgus monkeys; one untreated control animal also was used. Peritoneal lavage fluid, ovaries, oviducts, and the vagina with cervix were collected for analysis. These analyses showed no measurable translocation of talc from the site of deposition.

Similarly, Wehner et al. (1986) applied a 0.3 mL suspension of 125 mg of a neutron-activated cosmetic talc blend vaginally to six female cynomolgus monkeys for 30 consecutive days. Elemental composition was provided, but not particle size or shape. Another six cynomolgus monkeys served as untreated controls. Two days after the 30th injection, a peritoneal lavage was performed in the treated animals to capture talc particles that had possibly adhered to the peritoneal cavity. The ovaries, oviducts, uterus, vagina with cervix, and lavage fluid of these animals were then collected for γ-ray analysis. Only vaginal and cervix samples in the exposed monkeys contained talc, indicating that talc did not translocate to areas beyond the application site.

Henderson et al. (1986) exposed eight female Sprague Dawley rats to endometrial instillation of 250 μL of talc (100 mg/mL; 3:1 ratio of silicon to magnesium) suspended in phosphate-buffered saline (PBS) using a Portex catheter, while six rats were administered talc via vaginal instillation (259 µL of 100 mg/mL talc via syringe). Animals were sacrificed between 1 day and 49 days after exposure (endometrial instillation) or between 24 h and 4 days after exposure (vaginal). Talc particles were found in the ovaries of all animals that received intrauterine talc installations, regardless of duration of follow up. Regarding intravaginal talc exposure, talc was found in the ovaries of only the two animals that were sacrificed 4 days after exposure. No talc particles were found in the ovaries of animals intravaginally exposed and sacrificed 24 and 48 h after exposure.

Five small studies of human subjects (n = 5–25) detected talc (among other particles) in ovarian tissue of women with ovarian cancer (Henderson et al., 1971; Henderson, Hamilton, and Griffiths, 1979; Heller et al., 1996; McDonald et al., 2019; Johnson et al., 2020). In the lone study with unexposed controls, McDonald et al. (2019) indicated that particle counts were relatively low in the ovary (6–11 particles/histologic section), although the authors assert this may translate to a much larger exposure when calculated on a per-gram-of tissue basis. McDonald et al. (2019) also observed some talc particles in the fallopian tubes and ovaries of unexposed patients, and attributed these findings to earlier medical procedures (e.g., examinations performed while wearing powdered gloves) and/or “general living.” A study of women with benign ovarian lesions did not find an association between reported perineal talc use and ovarian talc particle burden, and noted that the particle counts varied substantially across the 24 participants (Heller et al., 1996). Because the fallopian tubes act as a peristaltic pump to move sperm up the reproductive tract, it has been proposed that this mechanism could be relevant for substances such as talc (Zervomanolakis et al., 2007; Zeller et al., 2018); however, there is no literature to support this hypothesis.

### Experimental animal studies of talc and reproductive cancers

Four studies evaluated talc carcinogenicity in rodents (Wehner, Zwicker, and Cannon, 1977; Wagner et al., 1979; NTP, 1993; Keskin et al., 2009). However, three of the four studies assessed toxicity related to whole body inhalation of talc, and the relevance to genital routes of exposure is limited for these studies. A detailed discussion of the results for the three sub-chronic and chronic rodent bioassays is included in our previous systematic review; overall, these studies provide limited evidence of lung carcinogenicity except at high, likely non-human relevant doses associated with particle overload conditions (Lynch et al., 2022). A brief discussion of the study findings relevant to perineal and vaginal talc exposure and female reproductive organ outcomes (Keskin et al., 2009) is provided below, followed by the quality evaluation results. Full quality evaluation results are presented in Supplementary Table S2.

#### Summary of study findings

Keskin et al. (2009) evaluated non-neoplastic and neoplastic changes in the reproductive organs of groups of seven Sprague Dawley rats intravaginally and perineally exposed to 100 mg of talc (unspecified source) in saline for 3 months, compared with unexposed and saline-only controls. No neoplastic or preneoplastic changes in the vulva, vagina, uterus, fallopian tubes or ovaries in exposed animals were reported, although there was evidence of foreign body reaction or infection. Specifically, the rats exposed to talc had significant increases in diagnoses of vulvovaginitis, endometritis, pelvic infection, ovarian infection, or salpingitis and tubal occlusion, effects that are not consistently associated with ovarian cancer. There were no significant differences in body weight comparing the talc-exposed rats to the controls.

#### Quality evaluation results

We classified Keskin et al. (2009) as medium quality overall, based on sufficient test design, outcome assessment and data analysis methods. The authors also properly incorporated a control group for comparison, and randomly allocated test animals into experimental groups to reduce potential bias. There was an explicit description of talc aerosolization. Furthermore, test animal characteristics, primarily species and strain, were described in detail, and experimental groups all were determined to have sufficient sample sizes and statistical power. While overall considered a medium-quality study, Keskin et al. (2009) was rated low quality with respect to characterizing the test substance, failing to report substance source and purity and providing insufficient detail regarding test design and exposure characterization. Additionally, the 3-month test period is shorter than the standard 2-year follow-up for chronic bioassay studies. Outcome assessment for these studies typically encompassed a full histological examination or autopsy of the relevant organs. Health outcomes that were unrelated to exposure also were assessed across studies to account for sources of attrition outside the outcomes of interest. Although this study was overall considered to be of medium quality, as discussed previously, there are some limitations.

#### Summary and conclusions for animal evidence

Animal evidence informing the potential association between talc and reproductive cancer is very limited. The single study with perineal talc application was judged to be of medium quality and reported no indication of carcinogenicity in any reproductive organs. Overall, there is ***no evidence*** that talc causes ovarian or other reproductive tumors in rodents after perineal exposure based on limited evidence–a single negative study utilizing a subchronic exposure duration. Other non-cancer potentially adverse effects were noted in this study, though the authors stated that these effects were not indicators of pre-cancerous lesions or associated with increased cancer risk.

### Mechanistic and mode of action information

Because it is inert, talc is unlikely to induce systemic toxicity or exert carcinogenicity via MOAs typical of many chemical carcinogens (i.e., direct damage to DNA). As with other poorly soluble low toxicity particles, one postulated potential carcinogenic MOA for talc is chronic inflammation, long-term tissue irritation and release of inflammatory chemokines and cytokines, and reactive oxygen species (ROS) formation. In sufficient quantities, ROS can cause secondary genotoxic effects (DNA damage), and potentially, tumor development (Kadry Taher et al., 2019). Another possible MOA stems from immune-related effects, such as via reduction of antibodies that target a transmembrane protein that is overexpressed in ovarian and other tumors (Cramer et al., 2005; Muscat and Huncharek, 2008). Talc itself was negative for direct mutagenicity in all available assays and this postulated MOA is not discussed further (Lynch et al., 2022).

### Potential mechanisms of carcinogenicity

#### Inflammation

Hamilton et al. (1984) administered asbestos-free talc (100 mg/mL) to the ovaries of female Sprague-Dawley via intrabursal injection for a duration of up to 18 months. Ovaries continued to produce normal levels of steroid hormones. Ovarian tissue was cystic in appearance due to bursal distension, and histological examination showed decreased ovarian tissue and some focal areas of papillary change in the surface epithelium of four of 10 animals. The authors indicated that changes could be direct effects of talc exposure; they could also have resulted from the high concentration of steroid hormones that accumulated in the ovarian bursa (Hamilton et al., 1984). In other words, steroid hormones, which, while present in normal physiological concentrations, were concentrated in the follicular fluid of the distended bursa and thus perpetually acting on steroid hormone receptors.

Yumrutas et al. (2015) incised the uterine horn of seven Wistar rats and administered a single dose of 100 mg/kg talc of unspecified purity. Animals were sacrificed 1 month later and antioxidants as well as various markers of oxidative stress were measured. *Gsr* and *Sod1* were statistically significantly increased in talc-exposed animals relative to controls; other markers were not statistically significantly altered. miRNA associated with both apoptosis and apoptosis inhibition (miR-98, mi-R15b, miR-34b, miR-21) were statistically significantly increased in talc-exposed rats. Critically, this study is considered unreliable because the control group had no incisions or sham exposures, so it is not possible to determine whether effects were independent of the stress induced from the administration route.

Fletcher et al. (2019) cultured epithelial ovarian cancer cells and normal human macrophages with talcum powder (Johnson & Johnson; 500 mg in 10 mL of dimethyl sulfoxide) and measured selected redox enzymes. There were significant and dose-dependent increases in inducible nitric oxide synthase (iNOS), nitrate/nitrite, and myeloperoxidase (MPO), and decreases in the antioxidant enzymes superoxide dismutase (SOD), catalase (CAT), glutathione peroxidase (GPX), and glutathione reductase (GSR) in all cells, but responses were stronger in the cancer cells. Talc exposure also was associated with increased inflammation as measured by tumor marker CA-125, relative to controls. Talc increased cell proliferation and decreased apoptosis in cancer cells, whereas the effect on normal cells was not significantly different than controls. This indicates that it is possible that while not likely to induce cancer, talc could be associated with cancer promotion; however, the dose (500 mg) used in this study is very high, and unlikely to be relevant to human exposures from personal cosmetic talcum powder application or occupational inhalation exposure to talc. Furthermore, whether changes to redox enzymes correlate with, much less directly predict, increased ovarian cancer risk is unknown. While some of these enzymes have been associated with certain cancer types, they likely are generic markers and not equally clinically relevant (Marrocco et al., 2017). Perhaps more importantly, ROS are detoxified by antioxidants, and given that there is a delicate balance of intracellular ROS and antioxidants, only when the balance is upset would cancer be initiated (Liou and Storz, 2010).

Mandarino et al. (2020) cultured phagocytic murine cells lines J774 and IC21 with 0.1–20 μg/well talc (<10 µm particle diameter, asbestos-free), suspended in phosphate buffered saline alone or with estradiol. Reactive oxygen species increased with talc exposure, particularly with estrogen co-exposure, and several genes associated with carcinogenic processes and with dampened immunosurveillance were upregulated, whereas cell number was unaffected. Talc also was co-cultured with murine ovarian surface epithelial cells (MOSEC), a “prototype for certain forms of ovarian cancer,” and phagocytosis was measured. Talc alone did not affect the number of MOSEC cells; however, phagocytosis was decreased in the presence of talc and estradiol, allowing for greater survival of MOSEC cells. The authors noted that the estradiol dose was likely high relative to normal mammalian endogenous levels, that the study was limited by the fact that they did not “investigate the carcinogenic properties of talc *per se*” and that additional studies would be needed to determine whether the effect of phagocytosis occurs *in vivo*, particularly in humans (Mandarino et al., 2020).

#### Immune response

Cramer et al. (2005) evaluated associations between ovarian cancer and the presence of epithelial mucin (MUC1) protein, present in numerous tissues. Ovarian and other cancers are associated with increased expression of MUC1. Specifically, authors quantified anti-MUC1 antibodies in women serving as controls in an ovarian cancer study (no cases were evaluated for anti-MUC1 antibodies). The authors reported that “38.1% of women who reported no use of cosmetic talc had antibody compared with 28.6% of women who regularly used talc” (*p* = 0.04); in tests of trend by talc use frequency (daily, weekly or less than weekly), there was a borderline significant (*p* = 0.11) association between genital talc use and decreased anti-MUC1 antibodies. In another study of ovulation, MUC1 and ovarian cancer, talc use was not associated with an increased number of ovulatory cycles or anti-MUC1 antibodies (Terry et al., 2007).

## Translocation and mode of action conclusion

Experimental animal studies of the translocation potential of talc within the reproductive tract after perineal/intravaginal exposure largely showed that talc did not move from the external genital area or vagina to the ovary. When talc was instilled directly into the uterus, talc was detected; however, this route is not relevant to human exposure conditions. Talc has been detected in women with ovarian cancer in a few small studies; however, the relatively low burdens of talc particles were not correlated with magnitude of exposure, and in one study talc particle burden coincided with the detection of numerous other particle types, including endogenous minerals.

The evidence regarding possible carcinogenic mechanisms of talc has been evaluated in a relatively small number of studies. A directly mutagenic MOA is not indicated when considering the negative results but perhaps more importantly, considering cosmetic and industrial talcs are chemically inert, poorly soluble, and fairly large particles (µm size range). Unfortunately, several of the available talc genotoxicity studies are low quality, employing methodologies now thought less informative for predicting genotoxicity (e.g., dominant lethal and unscheduled DNA synthesis [UDS] assays; see Zeller et al., 2018), and/or not providing the composition of the talc tested. While uncertainties remain, the overall weight of evidence is that talc is not expected to be directly mutagenic. Any genotoxic activity of talc would be expected to be secondary to oxidative DNA damage that results from ROS generated during particle-elicited inflammation, as seen for other poorly soluble particles (Schins and Knaapen, 2007).

Regarding the hypothesized MOA of chronic inflammation in the reproductive tract, studies reported epithelial changes *in vivo* and markers of inflammation and decreased phagocytosis in ovarian cells exposed to talc. These studies collectively provide evidence of some possible key events in the proposed inflammatory MOA; however, data are limited to non-human relevant exposure pathways and/or cell-based assays. There are no studies of key events that are further along the pathway to possible tumor formation after talc exposure. There is a delicate balance between ROS and antioxidants and only when protective mechanisms are overwhelmed will later effects on the pathway occur. This is an important data gap in the understanding of whether there is a plausible inflammatory MOA for talc and tumor formation (Marocco et al., 2017).

Another hypothesized MOA for talc and ovarian cancer is through decreased anti-MUC1 antibodies; however, the available information supporting this MOA is sparse. Overall, the mechanistic evidence is insufficient to support any MOA whereby talc can induce carcinogenesis in the female reproductive tract at human relevant exposure levels.

### Epidemiology

The number of published epidemiological studies on genital talc use and risk of reproductive cancers in women varies by cancer site, with some studies addressing multiple cancer outcomes. Based on the results of our search and preliminary review process described above, we determined that the largest number of studies addressed ovarian cancer, followed by endometrial and cervical cancers. Studies of other female reproductive cancers were addressed by few if any studies; therefore, we have limited our evaluation to ovarian cancer, including subtypes, and endometrial and cervical cancers.

### Ovarian cancer

**FIGURE 2:**
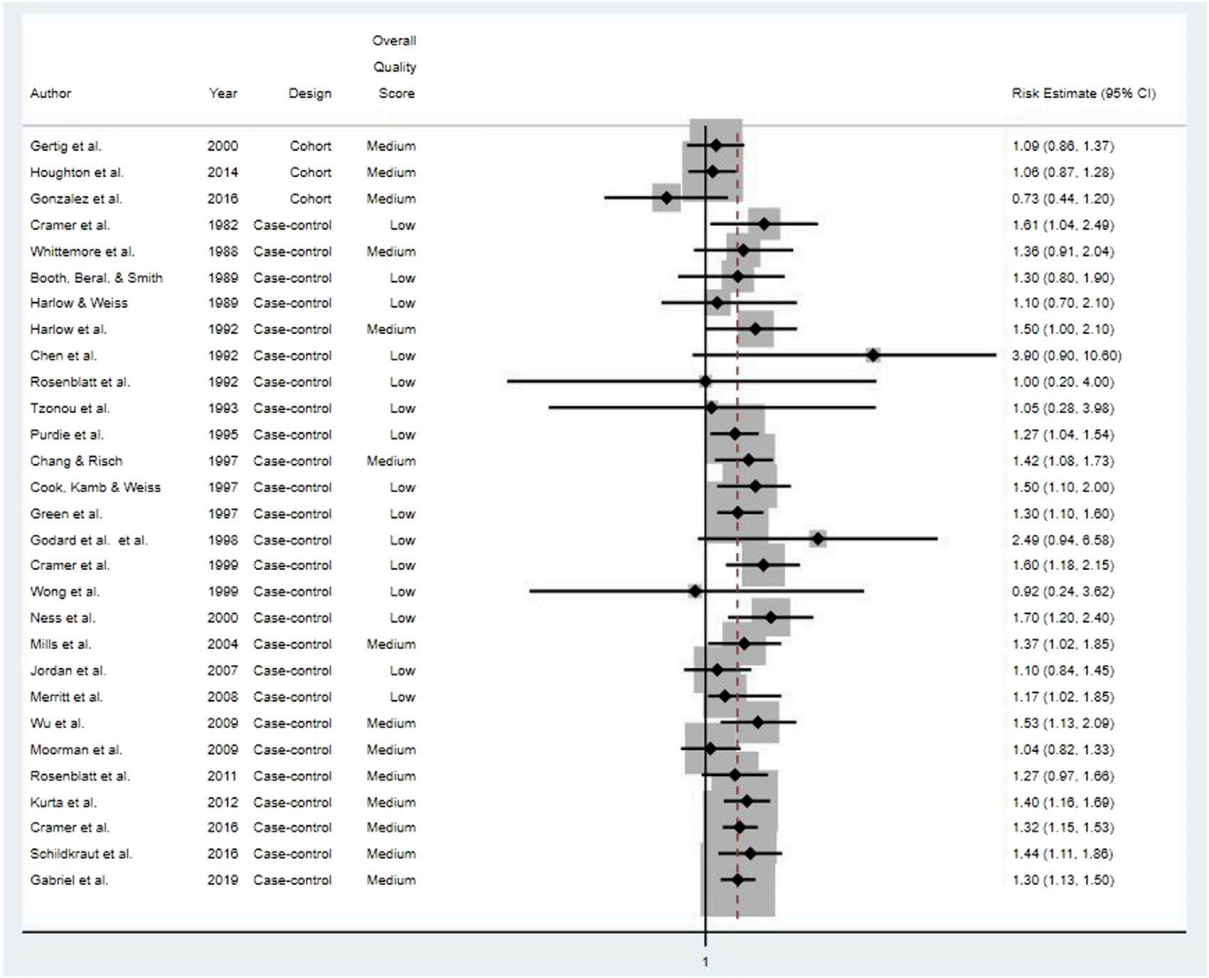
Risk estimates for all ovarian cancer types for studies of “any” genital talcum powder use.

We systematically reviewed the full-text publications on 31 studies that met study selection criteria and specifically examined potential associations between use of cosmetic talcum powders and ovarian cancer. Details on the study populations and methods for the five cohort studies, all prospective, and 26 case-control studies are presented in [Supplementary Table S4] and [Supplementary Table S3], respectively. Relative risk estimates for studies of “any” talc exposure and ovarian cancers (combined types) are provided in Figure 2. Due to key differences in study quality specific to study design, we summarize and discuss results from cohort and case-control studies separately below.

### Cohort studies—ovarian cancer

#### Results

None of the five prospective cohort studies reported any statistically significant associations between genital talcum powder use and risk of epithelial ovarian cancer, and relative risk estimates were close to unity (Gertig et al., 2000; Gates et al., 2010; Houghton et al., 2014; Urban et al., 2015; Gonzalez et al., 2016).

Two cohort studies reported no association between talc dusting of sanitary napkins and diaphragms and ovarian cancer (Gertig et al., 2000; Houghton et al., 2014). Frequency and duration of genital talc application also were not associated with increased ovarian cancer risk (Gertig et al., 2000; Gates et al., 2010; Urban et al., 2015).

The Nurses’ Health Study (NHS) cohort, comparing “ever” vs. “never” genital talc use, reported an elevated relative risk (RR = 1.40, 95% CI: 1.02–1.91) for invasive serous ovarian cancer after adjusting for several potential confounders (Gertig et al., 2000). Covariate-adjusted analyses evaluating talc use and risk of all serous ovarian cancers combined suggested a weaker association that was not statistically significant (RR = 1.26, 95% CI: 0.94–1.69). A 10-year update of the same cohort reported no association between genital talc use at least once per week and risk of serous invasive ovarian cancers (RR = 1.06, 95% CI: 0.84–1.34) detracting from the hypothesis that the initial observed association was related to talc use (Gates et al., 2010). Similarly, for the Women’s Health Initiative Observational Study (WHI-OS) cohort, Houghton et al. (2014) observed no evidence of an association between genital talc use and all serous ovarian cancers combined (hazard ratio [HR] = 1.16, 95% CI: 0.88–1.53) or with invasive serous ovarian cancer (HR = 1.13, 95% CI: 0.84–1.51).

#### Quality evaluation

**FIGURE 3:**
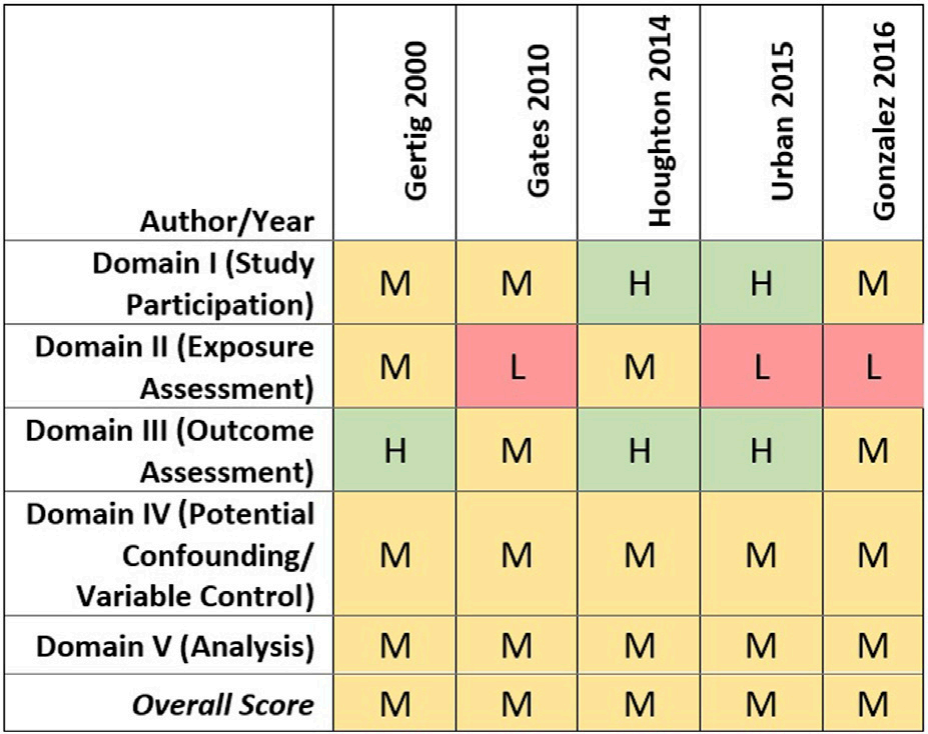
Heat map of study quality evaluation results for cohort studies of talc and ovarian cancer.

All cohort studies received a medium quality score overall, although there were differences in quality ratings across assessed domains (Gertig et al., 2000; Gates et al., 2010; Houghton et al., 2014; Urban et al., 2015; Gonzalez et al., 2016). In brief, most studies were rated medium quality based on the domains of participant selection, outcome assessment, and evaluation of potential confounding (see Figure 3). It should be noted, however, that covariate data were self-reported for all cohort studies, and three only assessed covariates at baseline, which would not capture any changes over time (i.e., parity, tubal ligation, oral contraceptive use, co-morbidities, and obesity) (Houghton et al., 2014; Urban et al., 2015; Gonzalez et al., 2016). Gertig et al. (2000) and Gates et al. (2010), however, re-assessed throughout follow-up covariates that may change over time and updated them as appropriate in their analyses.

Lower quality study ratings primarily were driven by the talcum powder perineal exposure characterization approach and exposure metrics used. Four of the cohort studies collected information on lifetime genital talc use as of baseline only (Gertig et al., 2000; Gates et al., 2010; Houghton et al., 2014; Urban et al., 2015).

Details regarding cohort members’ genital talc use were sparse. Gonzalez et al. (2016) compared those reporting “ever” to “never” genital talc use in the 12 months prior to enrollment and follow-up. Gates et al. (2010), Houghton et al. (2014), and Urban et al. (2015) obtained information on frequency and duration of talc use, allowing preliminary evaluation of possible exposure-responses. Houghton et al. (2014) captured duration of talc use, but reduced it in the analysis to a binary measure of less than 10 or 10 or more years of use. While Gertig et al. (2000) assessed genital talc use based on three frequency levels (i.e., less than once per week, 1–6 times per week, or daily use vs. never use), duration of use was not captured, precluding more precise exposure-response assessment including latency analyses should excess risks be observed. These limitations in exposure assessment resulted in lower confidence in the estimated exposure metrics for all five cohort studies.

Nevertheless, due to their prospective design, all of the cohort studies ascertained genital tac use among large numbers of women free of ovarian cancer and in advance of those diagnosed with ovarian cancer during follow-up, ensuring that the self-reporting of genital talc use could not be influenced by the ovarian cancer diagnosis.

### Case-control studies—ovarian cancer

#### Results

Of the 26 case-control studies evaluated, 18 (69%) reported at least one statistically significant odds ratio for ovarian cancer and genital talcum powder use (Cramer et al., 1982; Booth, Beral, and Smith, 1989; Harlow and Weiss, 1989; Harlow et al., 1992; Rosenblatt, Szklo, and Rosenshein, 1992; Purdie et al., 1995; Chang and Risch, 1997; Green et al., 1997; Cramer et al., 1999; Ness et al., 2000; Mills et al., 2004; Merritt et al., 2008; Wu et al., 2009; Rosenblatt et al., 2011; Kurta et al., 2012; Cramer et al., 2016; Schildkraut et al., 2016; Gabriel et al., 2019). There was little indication of any consistent relationship or trend with frequency, duration, or estimated cumulative talc exposure. The remaining eight studies reported no statistically significant odds ratios between ovarian cancer and genital talcum powder use (Whittemore et al., 1988; Chen et al., 1992; Tzonou et al., 1993; Cook et al., 1997; Godard et al., 1998; Wong et al., 1999; Jordan et al., 2007; Moorman et al., 2009).

Eight case-control studies evaluated genital talc use by histological subtypes of ovarian cancer, six of which reported statistically significant odds ratios for serous ovarian cancer (Cook, Kamb, and Weiss, 1997; Cramer et al., 1999; Mills et al., 2004; Merritt et al., 2008; Cramer et al., 2016; Gabriel et al., 2019), and one reported a statistically significant association with endometrioid ovarian cancer (Cramer et al., 2016). Four case-control studies evaluated possible associations between histological subtypes of ovarian cancer, including serous and endometrioid ovarian cancer, and duration, frequency, and exposure timing of talc use (compared to no use). No clear associations were seen between any of the genital talc exposure metrics and surrogates and any histological subtype of ovarian cancer (Chang and Risch, 1997; Merritt et al., 2008; Rosenblatt et al., 2011; Cramer et al., 2016).

Although generally weak associations between reported genital talc use and ovarian cancer were reported by more than half of the case-control studies, estimated odds ratios were inconsistent in terms of strength, precision, and talc exposure surrogate. The rest of the case-control studies reported no positive associations. Some studies reported associations with genital application of talc in general, or for dusting of sanitary napkins or underwear; however, few associations were seen for talc applied to diaphragms or cervical caps (where exposure presumably would be more likely internalized), and one (Cramer et al., 2016) reported a statistically significant inverse association with epithelial ovarian cancer (RR = 0.73, 95% CI: 0.57, 0.93) (Whittemore et al., 1988; Harlow and Weiss, 1989; Harlow et al., 1992; Rosenblatt, Szklo, and Rosenshein, 1992; Cook, Kamb, and Weiss, 1997; Ness et al., 2000; Wu et al., 2009; Rosenblatt et al., 2011; Cramer et al., 2016).

#### Quality evaluation

**FIGURE 4:**
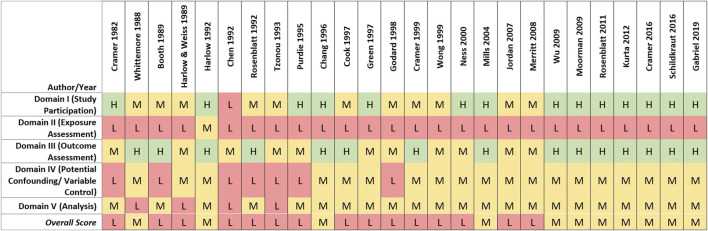
Heat map of study quality evaluation results for case-control studies of talc and ovarian cancer.

Of the 26 case-control studies assessed, none was rated high overall quality, 11 (42%) were rated medium, and 15 (58%) were rated low overall quality (see Figure 4). In most of the case-control studies, cases were identified from established registries or review of medical records, with histological confirmation via pathology reports. Studies receiving higher scores for study participation generally reported methods that indicated a low potential risk of selection bias. Medium and low study participation ratings primarily were driven by factors possibly increasing their susceptibility to selection biases, including differential recruitment methods, lack of reporting of baseline characteristics of cases and controls, or low or differential response rates between cases and controls.

Low overall quality scores for case-control studies primarily were driven by limitations in exposure assessment. Recall and self-reporting of historical talc exposure and other risk factors can be influenced by cases’ knowledge of their ovarian cancer diagnosis, especially if an association with a risk factor such as genital talc use is suspected or has been highlighted in the public media (Cramer et al., 1982). Such an effect was demonstrated by Schildkraut et al., 2016, where self-reported genital use of talcum powder was more strongly associated with ovarian cancer after litigation on this topic was amplified by national news media. As described in the methods section, lower exposure assessment ratings were assigned to case-control studies relative to cohort studies due to the high risk of recall and reporting bias. While talc use necessarily was assessed *post hoc*, many of the case-control studies attempted to characterize genital talc use by level, frequency or duration (n = 16, 61.5%). However, a substantial proportion of case-control studies (n = 10, 38.5%) only evaluated “ever” vs. “never” genital use of talcum powder, precluding evaluation of possible relationships with exposure characteristics or exploring indicators of recall or reporting bias (e.g., observing a general association with genital talc use that does not vary by amount, application type or timing of use, as generally observed in the studies that were able to perform relevant analyses).

Limitations related to the control of potential confounding factors or other analytical issues (e.g., missing or unclear descriptions of statistical methods and models) also contributed to lower-quality ratings (see Figure 4). As the recognized risk factors for ovarian cancer are more genetic and hormonal, potential confounders may not be easy to capture. Nevertheless, most studies reasonably obtained information on and considered potential confounders in the statistical analysis, resulting in medium overall scores for the potential confounding and variable control quality domain. Four studies received low ratings due to lack of sufficient statistical power and/or use of statistical models that were inappropriate or inadequately described (Whittemore et al., 1988; Harlow and Weiss, 1989; Chen et al., 1992; Tzonou et al., 1993).

#### Conclusions—ovarian cancer

Most case-control studies (n = 18) reported positive associations between ovarian cancer and genital talc use; however, the quality of the studies was mixed, with most studies assigned low-quality ratings specifically for exposure characterization and risk of bias. Among these studies, there were no consistent associations seen between ovarian cancer and reported talc application type, frequency, or duration of use; in other words, no consistent exposure-response relationships were identified in any studies.

Cohort studies that evaluated genital talc use by histological subtype of ovarian cancer reported no statistically significant associations for endometrioid, mucinous, ‘other’ ovarian cancer subtypes or invasive serous ovarian cancer (Gertig et al., 2000; Gates et al., 2010; Houghton et al., 2014; Gonzalez et al., 2016) (See Figure 3). Overall study quality also was mixed across the eight case-control studies that specifically evaluated serous ovarian cancer and genital talc use. Six studies of low and medium quality reported statistically significant associations, largely based on “ever” using talcum powder. In contrast, analyses of more precise indicators of talc use such as frequency, duration, and exposure timing did not identify clearer or more consistent associations with serous ovarian cancer. The two case-control studies that evaluated histological subtypes but did not identify a significant association between talc use and serous ovarian cancer (Chang and Risch, 1997; Rosenblatt et al., 2011) were rated as medium quality.

### Synthesis and conclusions for ovarian cancers

The cohort studies included in the systematic review consistently reported no associations between genital talc use and epithelial ovarian cancer overall, in contrast with the case-control studies, many of which reported inconsistent associations with the remaining studies reporting no statistically significant associations. For specific histologic types of ovarian cancer, a few studies reported associations with invasive serous ovarian cancer. However, except for one statistically significant relative risk estimate for ever use of genital talc and invasive serous ovarian cancer (Gertig et al., 2000) that was no longer apparent in a follow-up analysis (Houghton et al., 2014), the observed associations were of low magnitude and primarily derived from lower-quality case-control studies. More detailed exposure measures (e.g., frequency or duration of use) in cohort studies also tended not to be related to ovarian cancer (Gertig et al., 2000; Gates et al., 2010; Houghton et al., 2014).

Although application of talc to contraceptive diaphragms or cervical caps may represent a more direct route of exposure compared to external application or dusting, cohort and case-control studies evaluating such internal talc exposures failed to demonstrate any association with ovarian cancer. The only statistically significant association between talc use on diaphragms or cervical caps was a statistically significantly decreased relative risk of ovarian cancer (RR = 0.73, 95% CI: 0.57–0.93) (Cramer et al., 2016). Most of the cohort and case-control studies that evaluated talc application to diaphragms or cervical caps received a medium overall quality score (n = 6, 66.7%) (Whittemore et al., 1988; Harlow and Weiss, 1989; Wu et al., 2009; Rosenblatt et al., 2011; Houghton et al., 2014; Cramer et al., 2016). However, the numbers of women in these studies who applied talc to diaphragms or cervical caps were often small, resulting in imprecise relative risk estimates (Whittemore et al., 1988; Rosenblatt, Szklo, and Rosenshein, 1992; Wu et al., 2009).

Across both cohort and case-control studies, low-quality ratings primarily were driven by substantial limitations in the talc exposure assessment. Regardless of study design, studies assessing talc exposure at one time point via self-report are subject to inaccurate recall and reporting, leading to exposure misclassification; however, the expected direction and magnitude of such biases differ by study design. For cohort studies, participants reported genital talc use prior to ovarian cancer diagnosis, likely closer to the period of actual use and unaffected by any subsequent ovarian cancer diagnosis, thereby reducing potential bias whereas recall bias for case-control studies increases potential bias. Further, many cohort and case-control studies only or primarily analyzed ever talc use, which provided minimal information about possible changes in use patterns over time. These methods most likely result in random error, expected to be similar among women who subsequently develop ovarian cancer and those who do not. In contrast, case-control studies obtain information on talc use after cases have been diagnosed with ovarian cancer, and these women – especially if they believe that their cancer might have been caused by their genital talcum powder use–may better recall or possibly over-report their talc use compared with controls necessarily without ovarian cancer. Overall, more case-control studies included multiple surrogates of talc use including frequency and duration, than did the cohort studies; however, all such historical recall and reporting followed the ovarian cancer diagnosis, and required recall of use over previous decades rather than current or recent use.

Despite their limitations, the cohort studies on genital talc use and risk of ovarian cancer provide higher quality evidence overall compared to the case-control studies. Given the prospective design, inclusion of large national cohorts, and consistent medium quality ratings of the cohort studies (compared with a mix of medium and low ratings for the case-control studies), it is likely that the cohort studies collectively were better able to mitigate the effects of errors in recalling and reporting genital talc use, as these would not be expected to differ by any future cancer diagnosis.

Among the case-control studies assessed as medium quality, statistically significant associations between any talc use and epithelial ovarian cancer were more consistently reported in the recent studies, i.e., those published since 2012 (Kurta et al., 2012; Cramer et al., 2016; Schildkraut et al., 2016; Gabriel et al., 2019). The cases enrolled in these studies most likely would be aware of the widely publicized reported association between genital talc use and ovarian cancer risk, possibly contributing to recall and reporting bias in the case-control studies (Schildkraut et al., 2016; Casey and Larkin, 2019).

Despite the modest number of high-quality epidemiological studies addressing genital use of talc and ovarian cancer, the better-quality studies tend to be negative, providing insufficient evidence and an inadequate basis for concluding with any confidence that there is a causal connection. The several case-control studies reporting statistically significant associations between ovarian cancer and genital talc use all are overshadowed by recall and reporting bias, enhanced by the unavoidable exposure to news stories, social media and advertisements purporting that talcum powder causes cancer.

### Endometrial and cervical cancer

#### Introduction

We identified and systematically reviewed five publications evaluating potential associations between cosmetic talcum powder exposure and endometrial cancer, including four cohort studies (Karageorgi et al., 2010; Crawford et al., 2012; O’Brien et al., 2019; O’Brien et al., 2021) and one case-control study (Neill et al., 2012) [Supplementary Tables S5 and S6]. Only one publication was identified that evaluated cervical cancer (O’Brien et al., 2021).

Four studies utilized data from large, national cohorts, including the NHS (Karageorgi et al., 2010), the Sister Study (O’Brien et al., 2019; O’Brien et al., 2021), and the WHI-OS (Crawford et al., 2012). The one case-control study was conducted as part of the Australian National Endometrial Cancer Study (ANECS) (Neill et al., 2012). Across these studies, methods to ascertain talc exposure information and identify incident cancer cases were consistent with those described above regarding ovarian cancer. The case-control study conducted by Neill et al. (2012) included histologically confirmed endometrial cancer cases identified from treatment clinics and state-based cancer registries in Australia, as well as population-based controls matched on state and age-distribution. Specific endometrial cancer outcomes included overall endometrial cancer (Crawford et al., 2012); Type I endometrioid adenocarcinoma (Karageorgi et al., 2010); invasive endometrial cancers or adenocarcinomas (O’Brien et al., 2019); and Type I, Type II, and combined epithelial endometrial cancer (Neill et al., 2012).

#### Results

None of the four cohort studies and one case-control study reviewed that evaluated genital talc use and endometrial cancer reported a statistically significant overall association (Karageorgi et al., 2010; Crawford et al., 2012; Neill et al., 2012; O’Brien et al., 2019; O’Brien et al., 2021). Despite the majority of reported results across studies being null, three cohort studies reported at least one statistically significant relative risk estimate in sub-analyses by endometrial cancer type or by category of frequency or duration of talc use. However, there was no consistency in these statistically significant results, and as expected, some proportion (e.g., 5%) of all statistical tests performed at the alpha = 0.05 level will reflect chance associations. Furthermore, these relative risk estimates observed were generally weak and directionally inconsistent.

Crawford et al. (2012), amongst multiple comparisons by exposure type and duration, identified two statistically significantly increased relative risk estimates for endometrial cancer: using talc on a diaphragm for 20 or more years compared to none (HR = 3.06, 95% CI: 2.00–4.70) and using talc on a sanitary napkin for 10–19 years compared to none (HR = 1.63, 95% CI: 1.06–2.51). However, other durations of talc use on sanitary napkins or diaphragms, most notably including the longer use duration of 20 or more years on sanitary napkins, were not associated with endometrial cancer.

O’Brien et al. (2019) reported a statistically significantly increased relative risk for invasive endometrial cancer associated with ever talc use in nulliparous women compared to never use (HR = 2.1, 95% CI: 1.3–3.5). Karageorgi et al. (2010) identified an increased relative risk of endometrial cancer associated with regular perineal talc use when the analysis was restricted to postmenopausal women (RR = 1.24, 95% CI: 1.03–1.48), but similar associations were not seen for premenopausal women, when more detailed frequency measures were used (i.e., use less than once per week, 1–6 times per week, or daily vs. no use) or for the cohort overall. O’Brien et al. (2019) also evaluated ever- and last 12-month use of genital talc by menopausal status and reported no association with endometrial cancer in either pre- or post-menopausal women.

The case-control study by Neill et al. (2012) was the only study to evaluate frequency and duration of genital talc use, including categories of cumulative talc use (frequency x duration). No category of genital talc use was associated with epithelial endometrial cancer, regardless of talc use frequency, duration, or level. Conversely, the authors identified a negative association between long-term talc use and endometrial cancer, such that 61–80 years of talc use was strongly inversely associated with risk of epithelial endometrial cancer (OR = 0.25, 95% CI: 0.15–0.43), Type I (OR = 0.28, 95% CI: 0.16–0.49), and Type II (OR = 0.22, 95% CI: 0.07–0.67). Similarly, very high perineal talc use (40+ total talc-years) was inversely associated with epithelial endometrial cancer (OR = 0.67, 95% CI: 0.47–0.96). Regarding cervical cancer, O’Brien et al. (2021) observed no associations between talc use or frequency at ages 10–13 and pre-baseline cervical cancer, or between genital talc use in the past 12-month and cervical cancer incidence (O’Brien et al., 2021).

### Quality evaluation

Although quality domain scores varied across studies, all studies evaluating the potential association between genital talc use and endometrial cancer received a medium overall quality score (Karageorgi et al., 2010; Crawford et al., 2012; Neill et al., 2012; O’Brien et al., 2019). The one study of genital talc use and cervical cancer received a low overall quality score (O’Brien et al., 2021). See Supplementary Figures S3 and S4 for full study quality evaluation ratings.

The endometrial and cervical cancer studies generally used appropriate and reproducible analysis methods, including adjustment for potential confounders. Covariates, however, typically were self-reported, and only one cohort study re-assessed covariates throughout study follow-up (Karageorgi et al., 2010). Most studies used well-established methods to identify endometrial and cervical cancer cases, with cases initially identified via self-report subsequently confirmed by medical records and death certificates in cohort studies (Karageorgi et al., 2010; Crawford et al., 2012; O’Brien et al., 2019; O’Brien et al., 2021) and via clinics and statewide registries in the case-control study (Neill et al., 2012).

Consistent with the study quality patterns observed across the body of literature addressing ovarian cancer, lower quality ratings largely were driven by exposure characterization methods. Genital talc use was self-reported by participants at one time point in all studies, which could have resulted in inaccurate or biased reporting and misclassification of the exposure. Recall bias remains a particular concern for the one case-control study (Neill et al., 2012) and the cross-sectional evaluation of pre-baseline cervical cancer cases (O’Brien et al., 2021), as knowledge of their cancer diagnosis may have influenced cases’ historical recollection and reporting of genital talc use. Additionally, two studies attempted to evaluate genital talc use at ages 10–13 (O’Brien et al., 2019; O’Brien et al., 2021), which may be subject to inaccurate recall due to the specificity of the exposure period and length of time since respondents were pre-teenagers.

All studies included at least one talc exposure estimate with three or more levels, although the definition and level of detail varied. For example, Crawford et al. (2012) evaluated duration of genital talc use (<1 year, 1–4 years, 5–9 years, 10–19 years, 20+ years vs. never) and Karageorgi et al. (2010) evaluated ever perineal talc use (yes vs. no), frequency of perineal talc use (<once/week, 1–6 times/week and daily use), and regular perineal talc use (>once/week). Further, the temporal relationship between exposure and outcome was uncertain for the analysis of pre-baseline cervical cancer cases conducted by O’Brien et al. (2021), as well as the case-control study of endometrial cancer cases conducted by Neill et al. (2012), resulting in lower exposure characterization scores.

### Synthesis and conclusions for endometrial and cervical cancer

All endometrial studies reviewed were of similar quality overall with some differences in specific domains. No clear or consistent relationships were identified between genital talc use and endometrial cancer overall or by quality characteristics, although a few positive associations were reported (Crawford et al., 2012; Karageorgi et al., 2010; O’Brien et al., 2019. However, positive associations were primarily in subgroup analyses, inconsistent across studies, and based on limited talc use measures (i.e., ever vs. never use). The single case-control study identified a negative association, and evaluated more detailed talc use indicators (i.e., estimated frequency, duration, and level of lifetime talc exposure). The studies provide minimal information regarding endometrial cancer subtypes, and the heterogeneity in talc exposure indicators and endometrial cancer outcomes limit confidence in forming conclusions.

Regarding cervical cancer, evidence was available from only one study with low overall quality. There were no statistically significant associations identified between genital talc use and pre-baseline or incident cases. However, these results are limited by the significant limitations in exposure characterization, outcome assessment, and analysis methods.

Overall, the available evidence, while limited by the small number of studies and limitations in exposure, does not indicate any clear or consistent association between genital talc use and endometrial or cervical cancer.

### Evidence integration and hazard characterization

The basis for our conclusions regarding hazard for each cancer type is based on the IOM classification system (IOM, 2009) and described below as well as in the systematic review protocol. As discussed in the Methods, the IOM categorization offers the following categories:

⁃ Sufficient evidence of a causal relationship
⁃ Sufficient evidence of an association
⁃ Limited/suggestive evidence of an association
⁃ Inadequate/insufficient evidence to determine whether an association does or does not exist
⁃ Limited/suggestive evidence of no association

#### Ovarian cancer

One study in rats rated high quality reported on pure, micronized cosmetic talc and carcinogenicity. No ovarian tumors were observed in this study. With regard to mechanistic information, no translocation from perineal application to the ovaries was observed in several studies in rodents and monkeys. Human evidence is limited to a few studies detecting talc particles in the ovary; however, measured talc burden in the ovary was not associated with the magnitude of exposure (i.e., long-term/heavy talc use). Talc increased cell proliferation and decreased apoptosis in cancer cells and some normal cells *in vitro,* but study conditions (high dose, artificially high estradiol levels) were considered of limited relevance to *in vivo* exposures and to human exposures. The epidemiological evidence included numerous studies of ovarian cancer and genital talc use, but most of those reporting associations were case-control studies, most of which were considered of low quality primarily due to post-diagnosis historical self-reporting of talc use by women or even next-of-kin. Evidence of recall and reporting bias was greatest in the more recent studies after the possible association between talcum powder use and ovarian cancer risk was widely publicized. One meta-analysis reported an increased meta-RR estimate for invasive serous ovarian cancer, but risk was unrelated to various talc exposure indicators, suggesting that the overall association may not be due to talcum powder use (Berge et al., 2018). Goodman et al. (2003) demonstrated that serous ovarian cancer rates differed considerably by race, with rates among Whites nearly twice that of Blacks and Asian/Pacific Islanders. To the extent that genital application or reporting of genital application of talc differs by race has not been explored, and this could represent an important confounding factor. Despite the limited number of relevant, high-quality epidemiological studies, the subset of better-quality epidemiological studies provides insufficient evidence to conclude with any confidence that there is a causal connection. Integrating all lines of evidence, we therefore conclude that there is *suggestive evidence of no association* between genital talc use and ovarian cancer at human-relevant exposure levels.

#### Endometrial and cervical cancer

The body of evidence for endometrial and especially cervical cancer is somewhat sparse. However, study results from all lines of scientific inquiry largely mimic those for ovarian cancer. No endometrial or cervical tumors were observed in the sub-chronic animal study of perineal talc application. Mechanistic studies in animals indicate there may be some movement of talc from the external genital area to the cervix, but not into the uterus or beyond. No information was available regarding inflammatory or immune MOAs in endometrial or cervical cells or tissues. The five epidemiological studies evaluated, all of which were rated medium quality, demonstrated no association between genital talc use and endometrial or cervical cancer. Therefore, we again conclude that there is *suggestive evidence of no association* between genital talc use and endometrial cancer at human relevant exposure levels. Given the limited body of literature assessing the potential relationship between genital talc use and cervical cancer, we conclude there is *insufficient evidence to determine whether a causal association exists* for cervical cancer.

## Discussion

Our systematic review of talc and cancer finds *suggestive evidence of no association* for exposure to talc and ovarian cancer, as well as *suggestive evidence of no association* for endometrial cancer. We found *insufficient evidence to determine whether a causal association exists* for genital talc use and cervical cancer based on a limited body of literature. The body of epidemiological evidence is larger and more robust for ovarian cancer and provides critical information, complemented by high-quality experimental animal carcinogenicity bioassays and no convincing mechanistic evidence. Although the paucity of mechanistic information remains a limitation, the balance of evidence, especially the negative animal studies and body of higher-quality cohort studies demonstrating no increased cancer risks, provides the best scientific evidence of lack of carcinogenicity, at least at “real world” human-relevant exposures.

No prior reviews were identified on talc and endometrial or cervical cancers; however, a number of prior reviews have evaluated perineal talc use and ovarian cancer. Our systematic review is in general agreement with the critical review and weight-of-evidence assessment of ovarian cancer and talc exposure published by Goodman et al. (2020), which concluded that, “the available evidence does not support a causal association between talc and ovarian cancer.” As noted above, Berge et al. (2018) reported an increased meta-RR for the invasive serous type of ovarian cancer, but did not observe an association with any of the talcum powder exposure metrics. In contrast, reviews of the epidemiological evidence such as Wentzensen and O’Brien (2021) and Penninkilampi and Eslick (2018) concluded there is an association between perineal talc use and ovarian cancer, primarily based on findings from the case-control studies and the sub-group analysis reported in one cohort but was largely absent in an updated analysis (Gertig et al., 2000; Gates et al., 2010). Further, although Wentzsensen and O’Brien (2021) acknowledged associations, they ultimately concluded that “[g]iven the inability to attribute a clear causal factor to the observed associations, the lack of a good experimental model … and the inability to rule out confounding by indication, it is difficult to conclude that the observed associations are causal” (Wentzensen and O’Brien, 2021). O’Brien et al. (2023) evaluated the reliability of self-reported data on douching and genital talc use in the Sister Study and found that while women were relatively consistent in reporting their product use at enrollment relative to 10–14 years later, at least 10% of the population provided different answers about use across the survey period. In particular, self-report of genital talc use increased in women with an intervening ovarian cancer diagnosis, from 28% to 33% of the full sample. As such, the investigators suggested that recall bias was present and potentially contributed to the heterogenous effect estimates derived from case-control and cohort studies. Our study quality evaluation results reinforce this hypothesis given the consistent exposure assessment limitations observed within the case-control studies.

The comprehensive searches and detailed methods we followed are documented in this report and the Supplementary Materials so that others can verify, replicate and (hopefully constructively) comment on what we have done. Furthermore, our systematic review and integration of the scientific evidence on human cancer risks drew from the strongest aspects of established methodologies of several organizations’ systematic review guidance in an attempt to provide a full and transparent evaluation. We recognize, however, that there still may be areas of refinement in the approach. Further, while we attempted to highlight the most critical areas of study quality (for example, exposure characterization in epidemiological studies), some domains were uninformative (notably, the “analysis” domain). Additionally, because we did not use any tiering system for the quality evaluation of animal studies, the studies appear largely homogenous, when there might have been individual quality metrics that could be used to further differentiate the relative quality of these studies.

We believe that this comprehensive systematic review will be useful to researchers, regulators, policymakers, and other stakeholders concerned with the carcinogenicity of talc and talcum powder products under human-relevant exposure conditions, as well as help identify remaining research gaps. Nevertheless, based on the integration of evidence from animal experiments, mechanistic evaluations and epidemiological studies of reasonable methodological quality, this systematic review demonstrates that talc and cosmetic talcum powders unlikely cause female reproductive cancers at human-relevant exposures.
