# Supplementary material for: Systematic review of the association between talc and female reproductive tract cancers
Source: Front Toxicol. 2023 Aug 7;5:1157761. doi: 10.3389/ftox.2023.1157761 (PMC10442069; doi:10.3389/ftox.2023.1157761)
Supplement: Supplementary file 3 [file Table12.DOCX]

| **Table S.7. Evidence Integration Summary Judgment: Ovarian Cancer** | | | | | |
| --- | --- | --- | --- | --- | --- |
| **Summary of Animal, Human, and Mechanistic Evidence** | | | | | **Inference across evidence streams** |
| **Evidence from Studies of Exposed Humans** | | | | | *Suggestive Evidence of no association*   - Higher quality cohort studies largely null - Positive findings limited substantially by recall bias - No ovarian lesions or tumors in animal models   Other inferences:     - Several animal studies show no translocation of talc from perineum - Talc particle burden in humans not consistently associated with magnitude of talc use - Talc is not DNA reactive - Insufficient evidence supporting an MOA for ovarian carcinogenesis |
| **Studies, outcome and confidence** | **Key Findings** | **Factors that increase certainty** | **Factors that decrease certainty** | \| **Summary strength of evidence judgment** \| \| --- \| |  |
| **Ovarian cancer – Cohort Studies**  5 *medium* quality studies | - No significant association with epithelial ovarian cancer overall in 4 studies - Significantly increased risk of serous ovarian cancer (ever talc use) in 1 study | - Temporality established in prospective design - Large, national cohorts - Consistently null for epithelial ovarian cancer - Only significant risk estimate for serous was low magnitude, not replicated | - Self-reported talc use; limited measures for some analyses (ever v. never use) | Indeterminate |  |
| **Ovarian cancer – Case-control studies**  15 *low* quality studies  11 *medium* quality studies | - Significantly increased risk of overall epithelial ovarian cancer and subtypes in about half of the case-control studies | - More precise talc exposure measures in some studies (frequency or duration w/≥3 levels) | - No temporality - Increased likelihood of recall/reporting bias (self report after exposure) - Effect estimates of low magnitude - Lack of dose-response trends | Slight |  |
| **Evidence from *In Vivo* Animal Studies** | | | | |  |
| **Studies, outcomes, and confidence** | **Key Factors** | **Factors that increase certainty** | **Factors that decrease certainty** | **Summary strength of evidence judgment** |  |
| 4 *high-quality* studies in rats and mice | - No ovarian or other reproductive tract tumors - Lung tumors observed in one species in one of four studies | - Relatively high quality studies - Consistently null findings for the target organ of interest - Tumors found largely at doses exceeding MTD | - Carcinogenicity at other sites (lung, other tumors w/high spontaneous rates) | Evidence against |  |
| **Mechanistic Evidence or Supplemental Information** | | | | |  |
| **Biological events or pathways (or other information category)** | **Primary evidence evaluated** | **Key findings, interpretation, and limitations** | **Evidence stream summary** | |  |
| Talc translocation from external application into the reproductive tract | - 4 animal studies of intravaginal or intrauterine administration - 3 small studies of human talc ovarian burden | - Vaginal/perineal application in animals: no translocation to ovaries in monkeys, rats - Low levels of talc in ovaries of humans but few participants and no relation to duration/magnitude of exposure | - Animal studies indicate no substantial amounts of externally applied talc will reach the ovary - Human evidence of talc burden limited/not associated with usage patterns - Available mechanistic evidence insufficient to support any mode (or modes) of action for talc and reproductive cancers | |  |
| Carcinogenic Mechanisms:  Chronic Inflammation and genotoxicity | - 3 GLP/*guideline (K=1)* genotoxicity studies - 2 medium quality (K=2) *in vitro* mechanistic studies in normal and cancerous ovarian cells | - Not genotoxic - Causes inflammation - High cellular doses > exposure scenarios in humans - No *in vivo* studies of inflammation or immune-related mechanisms |  |  |  |
