# Supplementary material for: Systematic review of the association between talc and female reproductive tract cancers
Source: Front Toxicol. 2023 Aug 7;5:1157761. doi: 10.3389/ftox.2023.1157761 (PMC10442069; doi:10.3389/ftox.2023.1157761)
Supplement: Supplementary file 4 [file Table7.docx]

# **Table S.2 – Quality Evaluation Ratings for Animal Data**

# Keskin (2009)

| **Duration** | **Metric** | **Rating** | **Comments** |
| --- | --- | --- | --- |
| Test Substance | 1. Identity | 3 | Identified talc but no other details provided |
|  | 2. Source | 3 | Not reported |
|  | 3. Purity | 3 | Not reported |
| Test Design | 4. Negative and Vehicle Controls | 1 | 2 control groups, 1 with no intervention and 1 with saline |
|  | 5. Positive Controls | NR |  |
|  | 6. Assay Randomized Allocation | 3 | Not reported |
| Exposure Characterization | 7. Preparation and Storage of Test Substance | 3 | Not reported |
|  | 8. Consistency of Administration | 3 | Aerosolized talc applied across exposure groups but specifics not reported to allow for assessment of consistency |
|  | 9. Reporting of Doses/Concentrations | 2 | 100 mg in 0.5 mL saline but not mean or SD reported |
|  | 10. Exposure Frequency and Duration | 3 | Daily for 3 months (less than lifetime) |
|  | 11. Number of Exposure Groups and Dose Spacing | 2 | 4 exposure groups with different exposure types although rationale for spacing was not reported |
|  | 12. Exposure Route and Method | 1 | Aerosolized perineal and vaginal application |
| Test Organism | 13. Test Animal Characteristics | 3 | Animal characteristics were provided but source was not |
|  | 14. Adequacy and Consistency of Animal Husbandry Conditions | 2 | Light/dark cycle, food and water, and temperature provided |
|  | 15. Number per Group | 2 | 7 females per group |
| Outcome Assessment | 16. Outcome Assessment Methodology | 1 | Detailed histological examination |
|  | 17. Consistency of Outcome Assessment | 1 | Consistent for all animals |
|  | 18. Sampling Adequacy | 2 | Sampling number relatively low at 7 animals/group |
|  | 19. Blinding of Assessors | 2 | Not reported but unlikely to have an impact on outcome |
|  | 20. Negative and Control Response | 1 | Minimal level of inflammation relative to experimental groups |
| Confounding/Variable Control | 21. Confounding Variables in Test Design and Procedures | 1 | No difference in initial weight reported |
|  | 22. Health Outcome Unrelated to Exposure | 2 | No report of unrelated outcomes and impact on attrition although did not seem relevant |
| Data Presentation and Analysis | 23. Statistical Methods | 1 | Analysis using Fisher’s exact test |
|  | 24. Reporting of Data | 1 | Findings reported in detail for each group |
