# Supplementary figures and images for: Systematic review of the association between talc and female reproductive tract cancers
Source: Front Toxicol. 2023 Aug 7;5:1157761. doi: 10.3389/ftox.2023.1157761 (PMC10442069; doi:10.3389/ftox.2023.1157761)

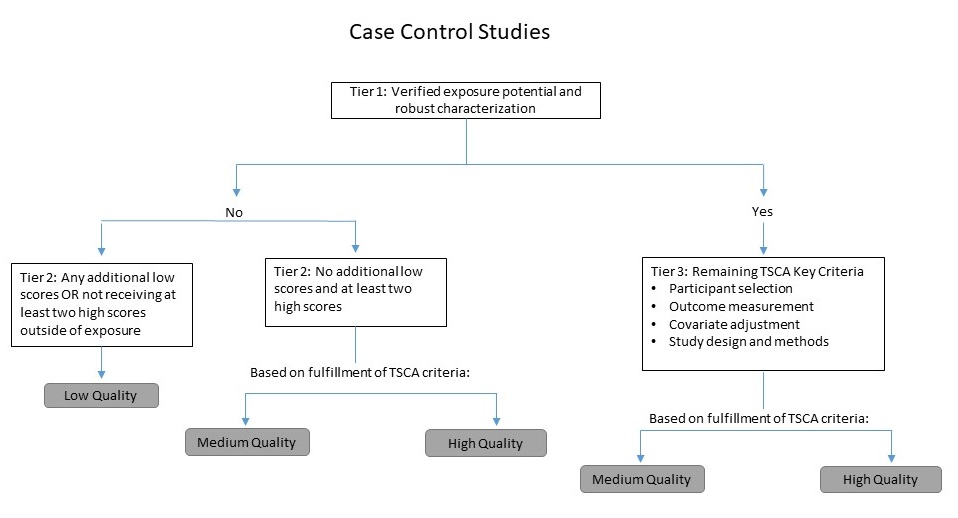


**Figure S.1. Study Quality Evaluation Approach – Case Control**

Supplement: Supplementary file 5 [file Table2.DOCX]

**
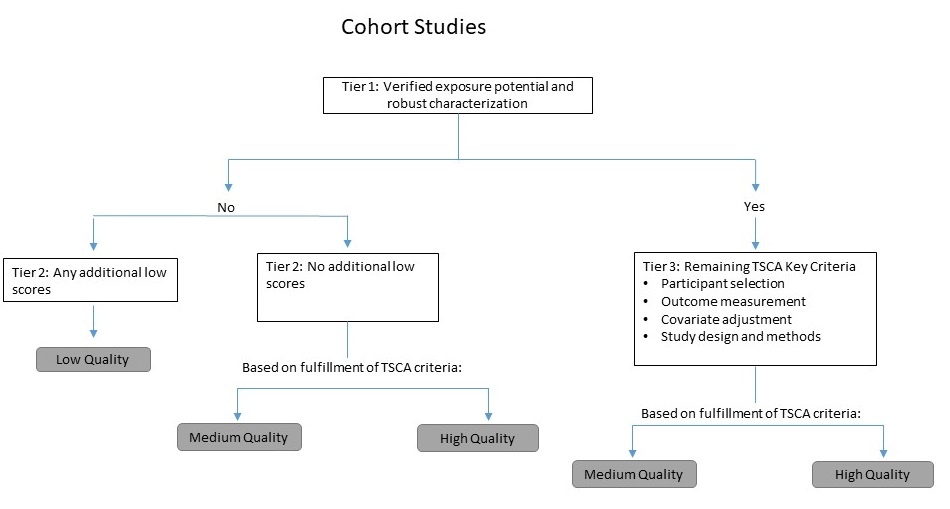
**

**Figure S.2. Study Quality Evaluation Approach – Cohort**

Supplement: Supplementary file 8 [file Table3.DOCX]
