# Supplementary material for: Systematic review of the association between talc and female reproductive tract cancers
Source: Front Toxicol. 2023 Aug 7;5:1157761. doi: 10.3389/ftox.2023.1157761 (PMC10442069; doi:10.3389/ftox.2023.1157761)
Supplement: Supplementary file 9 [file Table13.DOCX]

| **Table S.8. Evidence Integration Summary Judgment: Endometrial and Cervical Cancer** | | | | | |
| --- | --- | --- | --- | --- | --- |
| **Summary of Animal, Human, and Mechanistic Evidence** | | | | | **Inference across evidence streams** |
| **Evidence from Studies of Exposed Humans** | | | | | *Suggestive Evidence of no association (endometrial)*   - Higher quality cohort studies largely null - Positive findings limited substantially by recall bias - No uterine/cervical lesions or tumors in animal models   Other inferences:     - Several animal studies show little translocation of talc from perineum - Talc is not DNA reactive - Insufficient evidence supporting an MOA for ovarian carcinogenesis   *Insufficient Evidence to determine whether a causal association exists (cervical)*   - Null but very limited body of literature (single epidemiological study) - No uterine/cervical tumors in animals   Other Inferences:   - No evidence of translocation to cervix from perineum |
| **Studies, outcome and confidence** | **Key Findings** | **Factors that increase certainty** | **Factors that decrease certainty** | \| **Summary strength of evidence judgment** \| \| --- \| |  |
| *Four high-quality* cohort studies and one *low-quality* case control study | - No overall associations between talc and uterine or cervical cancer - Subgroup analyses identified at least one statistically significant but weak finding | - Relatively high quality cohort studies - Positive results largely limited to ever v. never talc use | - Recall bias likely in the case control study - Very few studies available; including a single study for cervical cancer | Limited evidence of no association |  |
| **Evidence from *In Vivo* Animal Studies** | | | | |  |
| **Studies, outcomes, and confidence** | **Key Factors** | **Factors that increase certainty** | **Factors that decrease certainty** | **Summary strength of evidence judgment** |  |
| 4 *high-quality* studies in rats and mice | - No uterine tumors - Lung tumors observed in one species in one of four studies | - Relatively high quality studies - Consistently null findings for the target organ of interest - Other tumors found largely at doses exceeding MTD | - Carcinogenicity at other sites (lung, other tumors w/high spontaneous rates) | Evidence against |  |
| **Mechanistic Evidence or Supplemental Information** | | | | |  |
| **Biological events or pathways (or other information category)** | **Primary evidence evaluated** | **Key findings, interpretation, and limitations** | **Evidence stream summary** | |  |
| Talc translocation from external application into the reproductive tract | - 4 animal studies of intravaginal or intrauterine administration | - Vaginal/perineal application in animals: no translocation to uterus in monkeys, rats; some translocation to cervix in monkeys | - Animal studies indicate no substantial amounts of externally applied talc will reach the uterus; some detection of talc in the cervix - Human evidence of talc burden limited/not associated with usage patterns - Available mechanistic evidence insufficient to support any mode (or modes) of action for talc and reproductive cancers | |  |
| Carcinogenic Mechanisms:  Chronic Inflammation and genotoxicity | - 3 GLP/*guideline (K=1)* genotoxicity studies - 2 medium quality (K=2) *in vitro* mechanistic studies in normal and cancerous ovarian cells | - Not genotoxic - Causes inflammation - High cellular doses > exposure scenarios in humans - No *in vivo* studies of inflammation or immune-related mechanisms |  |  |  |
