# Supplementary material for: Systematic review of the association between talc and female reproductive tract cancers
Source: Front Toxicol. 2023 Aug 7;5:1157761. doi: 10.3389/ftox.2023.1157761 (PMC10442069; doi:10.3389/ftox.2023.1157761)
Supplement: Supplementary file 10 [file Table4.docx]

**Figure S.3. Heat Map of Study Quality Evaluating results for Cohort Studies of Talc and Endometrial and Cervical Cancer**

| **Author/Year** | **Karageorgi 2010** | **Crawford 2012** | **O'Brien 2019** | **O'Brien 2021** |
| --- | --- | --- | --- | --- |
| **Domain I (Study Participation)** | H | H | H | H |
| **Domain II (Exposure Assessment)** | M | M | L | L |
| **Domain III (Outcome Assessment)** | H | H | M | M |
| **Domain IV: Potential Confounding/ Variable Control** | M | M | L | L |
| **Domain V: Analysis** | M | M | M | M |
| **Overall Score** | M | M | L | L |
