# Supplementary material for: Systematic review of the association between talc and female reproductive tract cancers
Source: Front Toxicol. 2023 Aug 7;5:1157761. doi: 10.3389/ftox.2023.1157761 (PMC10442069; doi:10.3389/ftox.2023.1157761)
Supplement: Supplementary file 11 [file Table6.docx]

# **Table S.1 – Animal Data Extraction Results**

| Full Citation | Type of Talc | Talc Content (%) | Other Mineral Content (%) | Other Notes on Talc Under Study | Animal Model | Route of Exposure | Exposure Concentration | Exposure Duration | Type of Assays Performed | Significant Response Above Control (Y/N) | No. of Mesotheliomas vs. Controls | No. of Lung Cancers vs. Controls | No. of Fibrotic Disease vs. Controls | Significant Effects |
| --- | --- | --- | --- | --- | --- | --- | --- | --- | --- | --- | --- | --- | --- | --- |
| Keskin, N., Y. A. Teksen, E. G. Ongun, Y. Ozay, and H. Saygili. 2009. Does long-term talc exposure have a carcinogenic effect on the female genital system of rats? An experimental pilot study. Arch Gyn Obstet 280 (6):925-931. | Only stated "talc" | Not listed | Not listed | Not listed | Rats | Intravaginal and perineal aerosol application | 100 mg in 0.5 mL saline | Daily for 3 months | Histological Assessment | N | 0 | 0 | 0 | There was no evidence of neoplastic or preneoplastic change in any of the experimental groups although there was evidence of foreign body reaction/infection. |
